# MEMS Vibrometer for Structural Health Monitoring Using Guided Ultrasonic Waves

**DOI:** 10.3390/s22145368

**Published:** 2022-07-19

**Authors:** Jan Niklas Haus, Walter Lang, Thomas Roloff, Liv Rittmeier, Sarah Bornemann, Michael Sinapius, Andreas Dietzel

**Affiliations:** 1 Institute of Microtechnology, Technische Universität Braunschweig, 38124 Braunschweig, Germany; a.dietzel@tu-braunschweig.de; 2 Institute for Microsensors, Actuators and Systems, University of Bremen, 28359 Bremen, Germany; wlang@imsas.uni-bremen.de (W.L.); sbornemann@imsas.uni-bremen.de (S.B.); 3 Institute of Mechanics and Adaptronics, Technische Universität Braunschweig, 38106 Braunschweig, Germany; thomas.roloff@tu-braunschweig.de (T.R.); l.rittmeier@tu-braunschweig.de (L.R.); m.sinapius@tu-braunschweig.de (M.S.)

**Keywords:** MEMS vibrometer, structural health monitoring (SHM), guided ultrasonic waves (GUW), fiber metal laminates (FML)

## Abstract

Structural health monitoring of lightweight constructions made of composite materials can be performed using guided ultrasonic waves. If modern fiber metal laminates are used, this requires integrated sensors that can record the inner displacement oscillations caused by the propagating guided ultrasonic waves. Therefore, we developed a robust MEMS vibrometer that can be integrated while maintaining the structural and functional compliance of the laminate. This vibrometer is directly sensitive to the high-frequency displacements from structure-borne ultrasound when excited in a frequency range between its first and second eigenfrequency. The vibrometer is mostly realized by processes earlier developed for a pressure sensor but with additional femtosecond laser ablation and encapsulation. The piezoresistive transducer, made from silicon, is encapsulated between top and bottom glass lids. The eigenfrequencies are experimentally determined using an optical micro vibrometer setup. The MEMS vibrometer functionality and usability for structural health monitoring are demonstrated on a customized test rig by recording application-relevant guided ultrasonic wave packages with a central frequency of 100 kHz at a distance of 0.2 m from the exciting ultrasound transducer.

## 1. Introduction

Guided ultrasonic waves (GUW) can be used in structural health monitoring (SHM) [1]. Surface-mounted, usually piezoelectric, transducers are used to emit ultrasound bursts into the structure. The propagation of the GUW is then monitored by a sensor network to detect, localize, and quantify structural damage which locally changes the acoustic impedance, leading to reflections and possibly to mode conversions [2]. Piezoelectric [3,4] and capacitive [5] micromachined ultrasound transducers are currently being developed, but are so far only considered for recording acoustic emission and not designed to detect GUW. In these works, multiple element sensor chips for multi-frequency sensitivity are investigated.

Fiber metal laminates (FML) are being researched and partially already used in aerospace, as they combine the high specific strength of fiber composites with the ductile properties of metals. In FML, due to the high impedance differences between the metal layers and the layers of fiber-reinforced plastic, the wave propagation information is relevant to SHM mainly in the inner material layers. Therefore, sensor systems are required that can be embedded in the material with minimal retroactive effect on the propagation of GUW. Current research is concerned with integrated polyvinylidene fluoride (PVDF) film sensors in glass fiber reinforced polymer (GFRP) [6] and with lead zirconate titanate (PZT) transducers in carbon fiber reinforced polymer (CFRP) [7,8] for impact and fatigue sensing. New concepts for stretchable and integrable sensor networks of PZT sensors have been developed but only applied on a CFRP structure without being used for sensing GUW [9].

Piezoelectric transducer patches are used to sense propagating GUW based on the resulting strain, but typically need to cover at least half a wavelength of the ultrasound to operate efficiently [2,10]. In addition, the acoustic properties of piezoceramics are poorly adapted to the laminate material, resulting in a discontinuity of the acoustic impedance. Inertial MEMS (micro electro-mechanical system) sensors, in contrast, are based on excitation in the form of displacements of the housing and can therefore be miniaturized to the technical limits. Moreover, glass as a typical MEMS housing material is acoustically much better adapted to the polymer of FML [11].

However, typical MEMS accelerometers, such as those used for low-frequency SHM in civil engineering structures, have a bandwidth of a few kHz, which is far below ultrasound. Examples are the Analog Devices ADXL 345 with 3200 Hz [12] or the ST AIS2IH with 1600 Hz [13]. While accelerometers are sensitive to accelerations at frequencies well below the resonance, seismometers become more sensitive for displacements at frequencies above the fundamental resonance frequency [14]. This makes the seismometer concept more suitable for the detection of GUW. Certainly, out-of-plane motions at ultrasound frequencies can also be detected with optical vibrometers such as the laser Doppler vibrometer (LDV). However, unlike these optical techniques, which only have access to the visible surface of a structure, an MEMS sensor can be embedded to capture information from inside a structure. Therefore, the concept of an MEMS seismometer as embeddable vibrometer for the detection of narrow-band bursts, as used for active SHM using GUW, was recently proposed by the authors [11]. A GUW detection scheme based on this concept requires a sealed MEMS vibrometer that can be integrated into the layered structure of FML without perturbing the propagating GUW. This article presents such a concept.

## 2. The Concept of MEMS Vibrometer Response

**Figure 1:**
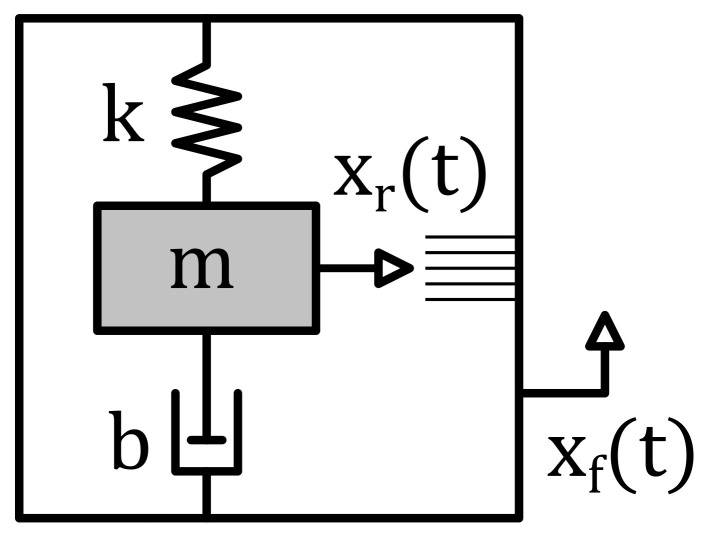
Schematic illustrating the inertial sensor concept [15].

Inertial sensors detect the relative displacement $x_{r}\left(t\right)$ of a spring-loaded mass *m* to the sensor frame position $x_{f}\left(t\right)$, which follows the external mechanical stimulus, as illustrated in Figure 1.

The well-known governing equation for such a foot point excited system is

(1) $$m\;\cdot \;\frac{d^{2}x_{r}\left(t\right)}{dt^{2}}+b\;\cdot \;\frac{dx_{r}\left(t\right)}{dt}+k\;\cdot \;x_{r}\left(t\right)=-m\;\cdot \;\frac{d^{2}x_{f}\left(t\right)}{dt^{2}},$$

where *m* represents the mass, *k* the spring constant, and *b* the damping. Such a system is characterized by the natural frequency $\omega_{0}$

(2) $$\omega_{0}=\sqrt{\frac{k}{m}}.$$

Assuming harmonic stimuli and responses, this allows the complex frequency response to an acceleration ${\underline{G}}_{accel}$ to be expressed as

(3) $${\underline{G}}_{accel}\left(i\omega \right)=\frac{-m}{{\left(i\omega \right)}^{2}\;\cdot \;m+i\omega \;\cdot \;b+k}$$

and the complex frequency response to the displacement ${\underline{G}}_{displ}$ as

(4) $${\underline{G}}_{displ}=\frac{-{\left(i\omega \right)}^{2}\;\cdot \;m}{{\left(i\omega \right)}^{2}\;\cdot \;m+i\omega \;\cdot \;b+k}=-\omega^{2}\;\cdot \;{\underline{G}}_{accel}.$$

**Figure 2:**
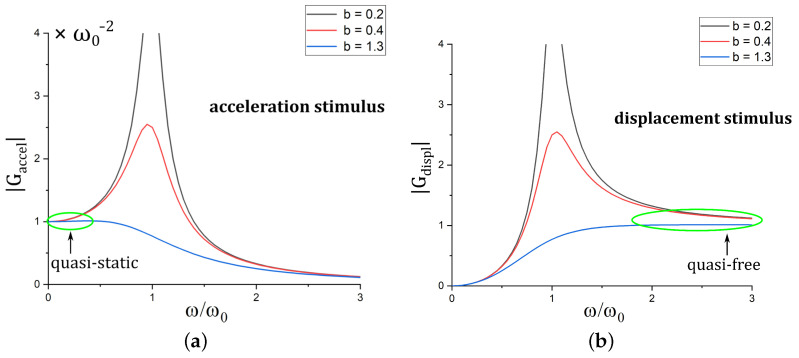
Frequency -dependent sensitivity to acceleration $|{\underline{G}}_{accel}|$ (**a**) and sensitivity to displacement $|{\underline{G}}_{displ}|$ (**b**). The colors indicate different levels of damping.

In the quasi-static frequency range, where $\omega \ll \omega_{0}$, the sensors sensitivity to acceleration is given as $|{\underline{G}}_{accel}|\approx \frac{1}{\omega_{0}^{2}}$ and the sensitivity to displacement as $|{\underline{G}}_{displ}|=\omega^{2}\;\cdot \;\left|{\underline{G}}_{accel}\right|\approx 0$ (cf. Figure 2). For the case of quasi-free excitation, i.e., where $\omega \gg \omega_{0}$, sensitivities are given as $|{\underline{G}}_{accel}|\approx \frac{1}{\omega^{2}}\approx 0$ and $|{\underline{G}}_{displ}|=\omega^{2}\;\cdot \;\left|{\underline{G}}_{accel}\right|\approx 1$. As a consequence, the spring-loaded mass acts as an accelerometer for $\omega \ll \omega_{0}$ and as a displacement sensor, also called seismometer, for $\omega \gg \omega_{0}$.

In the case of an LDV, the displacement must be determined from the measured vibration velocity, i.e., by integration. Any other device, which is also able to record the local vibrations with sufficient temporal resolution, can be referred to as a vibrometer. Here, an MEMS vibrometer (whose sensitivity is given by ${\underline{G}}_{displ}$) utilizing a micro cantilever as a key element shall be embedded in the FML structure to directly record the local displacements forced by the propagating GUW.

A cantilever is described by the spring-loaded mass theory only in approximation, as long as no higher-order resonance frequencies are considered. Due to the micro cantilever’s geometry, however, it behaves more complex than the model of a simple spring-mass damper system. In addition to $\omega_{0}$, higher eigenfrequencies, each corresponding to a bending or torsion mode, appear. For the operation as a vibrometer, the detectable frequencies must be in a spectral range, where $|{\underline{G}}_{displ}|\approx 1$, as is given in between the lowest (first) eigenfrequency $\omega_{0}$ and the second eigenfrequency of the cantilever. The bending mode eigenfrequencies $\omega_{i}$ of a cantilever are obtained as solutions of Equations (5) and (6) [16,17,18].

(5) $$\cos\lambda_{i}\;\cdot \;\cosh\lambda_{i}=-1$$

(6) $$\omega_{i}=\lambda_{i}^{2}\;\cdot \;\sqrt{\frac{E\;\cdot \;t^{2}}{12\;\cdot \;\rho \;\cdot \;L^{4}}}$$

Taking the geometry of a laser-cut cantilever (L=540 μm, t=10 μm) with the material properties of silicon ($E_{<100>}=165.6\;\times \;10^{9}\;N\;m^{-2}$, density $\rho =2330\;kg/m^{3}$), the bending eigenfrequencies are predicted as

$$f_{0}=47\;\mathrm{kHz};f_{1}=292\;\mathrm{kHz};f_{2}=818\;\mathrm{kHz};f_{3}=1.60\;\mathrm{MHz}.$$

These eigenfrequencies will only be considered as a first approximation for the physical resonator to be described in the following. The analytical prediction of torsional modes is rather complex. Therefore, eigenfrequencies are obtained experimentally, as will be described in Section 4.1.

## 3. Materials and Methods

### 3.1. Resonator Design and Fabrication

**Figure 3:**
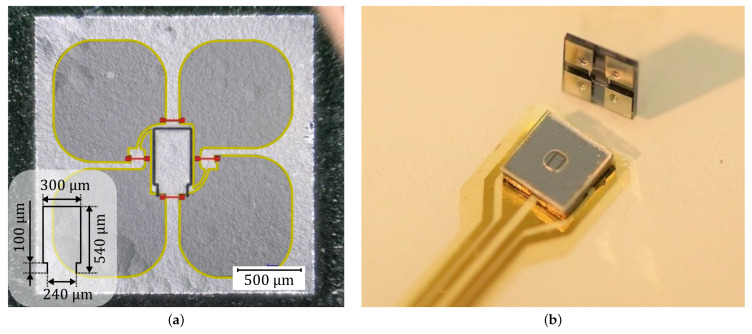
(**a**) Microscopic image of the chip surface after step (g) showing the femtosecond laser cuts in the silicon membrane and the Wheatstone bridge doping scheme. Dark gray areas marked by yellow frames indicate the highly-boron-doped silicon wiring; the four much smaller piezoresistive tracks with weaker doping are marked in red color. (**b**) MEMS vibrometers with 2 mm × 2 mm lateral dimensions and a thickness of 410 µm. One is mounted onto polyimide PCB substrate by reflow soldering.

In order to demonstrate the concept of an MEMS vibrometer, a micro cantilever is realized by laser-modifying the deformation-sensitive silicon membrane of an existing cavity-in-glass pressure sensor [19]. The cutting contour for the micro cantilever is dimensioned to obtain a large seismic mass and a low spring force within the limits in which the sensor’s Wheatstone circuit remains intact. It consists of four doped piezoresistors and a silicon wiring (cf. Figure 3a). By the laser contouring, it becomes sensitive to the deflection of the cantilever. For encapsulation, a glass lid with a cavity is bonded to the flat silicon surface to ensure free oscillation of the cantilever when embedded.

**Figure 4:**
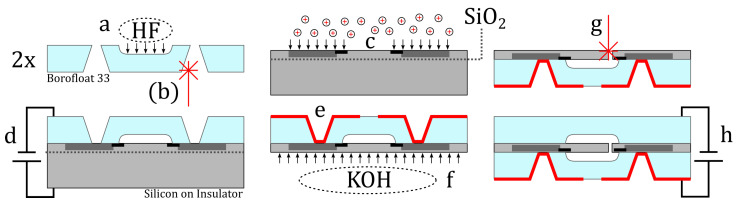
Illustration of the MEMS vibrometer microfabrication: (**a**) HF cavity etching into borofloat wafer; (**b**) through-glass vias by fs-laser ablation; (**c**) local boron-doping of the SOI device layer; (**d**) anodic bonding of SOI and borofloat wafers; (**e**) metallization by Cr+Au magnetron sputtering and successive Cu electroplating; (**f**) removing handle layer by KOH etching; (**g**) fs-laser cutting cantilever into membrane; (**h**) encapsulation by anodically bonding a glass lid.

The MEMS vibrometer fabrication using most of the process steps previously applied for the cavity-in-glass pressure sensor is illustrated in Figure 4. A 200 μ m thick borosilicate glass wafer with hydrofluoric acid (HF)-etched cavities (a) and femtosecond laser (λ=1030 nm) made through holes (b) and the device layer of silicon on insulator (SOI) wafer which is boron-doped (c) with a scheme that will later yield a Wheatstone bridge are anodically bonded (d). Magnetron sputtering of chromium and gold, and successive copper electroplating (e), yield through-glass vias from the silicon Wheatstone circuit to a solderable chip-scale package, providing mechanical and electrical connection to a printed circuit board (PCB) substrate. The SOI’s device layer forms a highly consistent 10 μm thick membrane after removing the handle layer by KOH etching (f). The 4-inch wafer is diced into 552 MEMS vibrometer chips. In order to create the micro cantilever, the membrane is modified on chip level by femtosecond laser ablation. A contour is cut (g) into the membrane, thereby creating a cantilever with approximate measures of 540×240−300×10 μm. To encapsulate the micro cantilever, a diced chip from a second borosilicate wafer with HF-etched cavities (a) is anodically bonded (h) to the MEMS silicon device layer, encapsulating an atmosphere with an absolute pressure of about $10^{5}\;N\;m^{-2}$ at $20\;{}^{\circ }C$. Finally, the obtained MEMS vibrometer is mounted to a 25 μm thin flexible polyimide PCB (see Figure 3b). Production steps (a)–(f) are detailed in [19]. The parameters for fs-laser membrane cutting (g) using a laser micromachining workstation (microSTRUCT C, 3D Micromac, Chemnitz, Germany, equipped with a [Yb:KGW] femtosecond laser source, PHAROS from Light Conversion, Vilnius, Lithuania and a laser scanner Intelliscan 14, Scanlab GmbH, Puchheim, Germany with a f=100 mm telecentric FTheta lens, Linos AG, Göttingen, Germany) are wavelength = 1030 nm, repetitions = 40, pulse energy = 7.7 μJ, pulse duration = 224 fs, scan speed = 500 mm/s, repetition rate = 599 kHz, and spot diameter = 25 μm. The anodic bonding parameters for the lid (h) are similar to (d) except for raising the bonding voltage to 1150 V to compensate the roughness by laser structuring. Metallized vias enable the electrical connection between the silicon and the anode (bond chuck).

### 3.2. Experimental Modal Analysis of the MEMS

**Figure 5:**
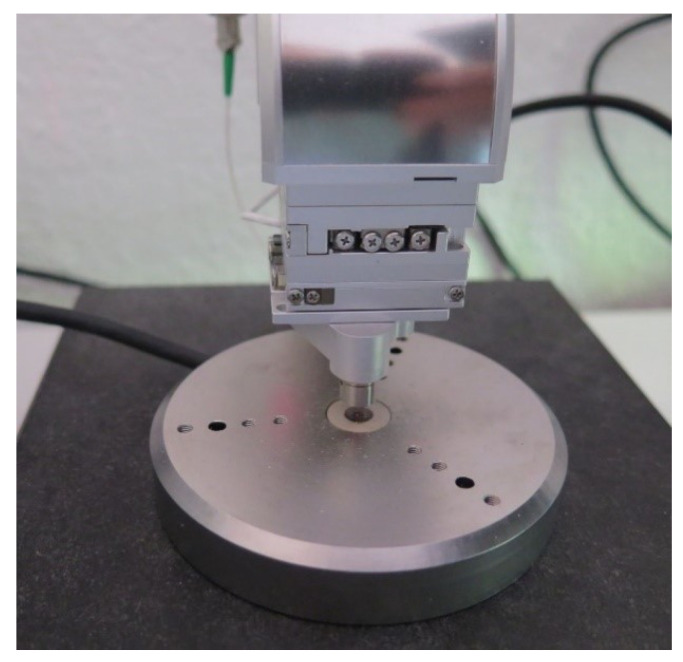
Measurement setup: the vibrometer head from the PicoScale vibrometer is raster-scanned over the sample, fixated onto the shaker stage for the dynamic characterization of the MEMS vibrometer chip.

To gain a better understanding of the sensor’s dynamic behavior, the eigenfrequencies and the corresponding modal shapes of the inherent micro cantilever are experimentally determined. The test setup (cf. Figure 5) comprises a scanning laser vibrometer that integrates a confocal microscope and a shaker stage for the high-frequency out-of-plane excitation with nanometer-scale amplitudes (PicoScale Vibrometer, SmarAct Metrology, Oldenburg, Germany). The MEMS vibrometer chip is mechanically coupled to the shaker stage using vacuum grease. After imaging the region of interest, single point vibrometry is performed at a corner of the cantilever tip, allowing to monitor bending and torsional modes. A frequency sweep with constant amplitude is used as excitation signal. For the imaging of the cantilever vibrational modes, the sample is excited with a harmonic sinusoid. Then, the measurement laser is raster-scanned over the sample and the obtained displacement data are fed into the internal digital lock-in amplifier, so that for each pixel the amplitude and phase of the vibration can be determined. With these data, the cantilever mode shapes can be directly observed.

### 3.3. Setup for Dynamic Sensor Signal Response Characterization

**Figure 6:**
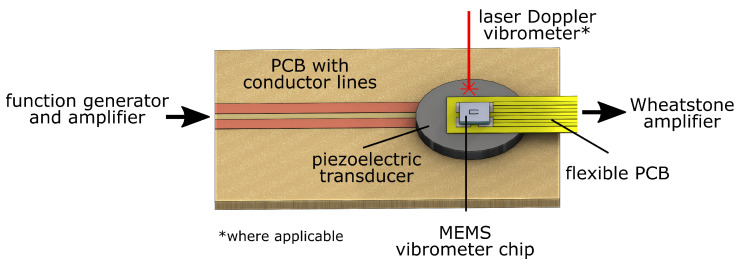
3D sketch of the test bed for the dynamic sensor response characterization.

The test bed shown in Figure 6 allows to characterize the electrical sensor response to ultrasonic structural displacements. A piezoelectric transducer (PRYY+0226, PI Ceramics, Lederhose, Germany) with a diameter of 10 mm, a thickness of 0.5 mm, and with wrap-around electrodes is soldered to a PCB substrate, yielding a simplified shaker stage. On top of it, the flexible polyimide PCB on which the chip-scale MEMS vibrometer is mounted is adhesively bonded. Using a function generator and a high-frequency voltage amplifier (PD200, PiezoDrive, Shortland, Australia), the shaker stage can be excited with arbitrary signals. Simultaneously, the output voltage of the MEMS vibrometer is recorded using a high-frequency Wheatstone bridge amplifier (DEWE 30-40, DEWETRON, Grambach, Austria). As a reference, the spot of an LDV (PSV 400, Polytec, Waldbronn, Germany) is directed to the chip transducer, directly next to the MEMS vibrometer chip. Using a sweep excitation from 0–500 kHz, the MEMS vibrometer’s transfer behavior can be derived.

### 3.4. GUW Setup

**Figure 7:**
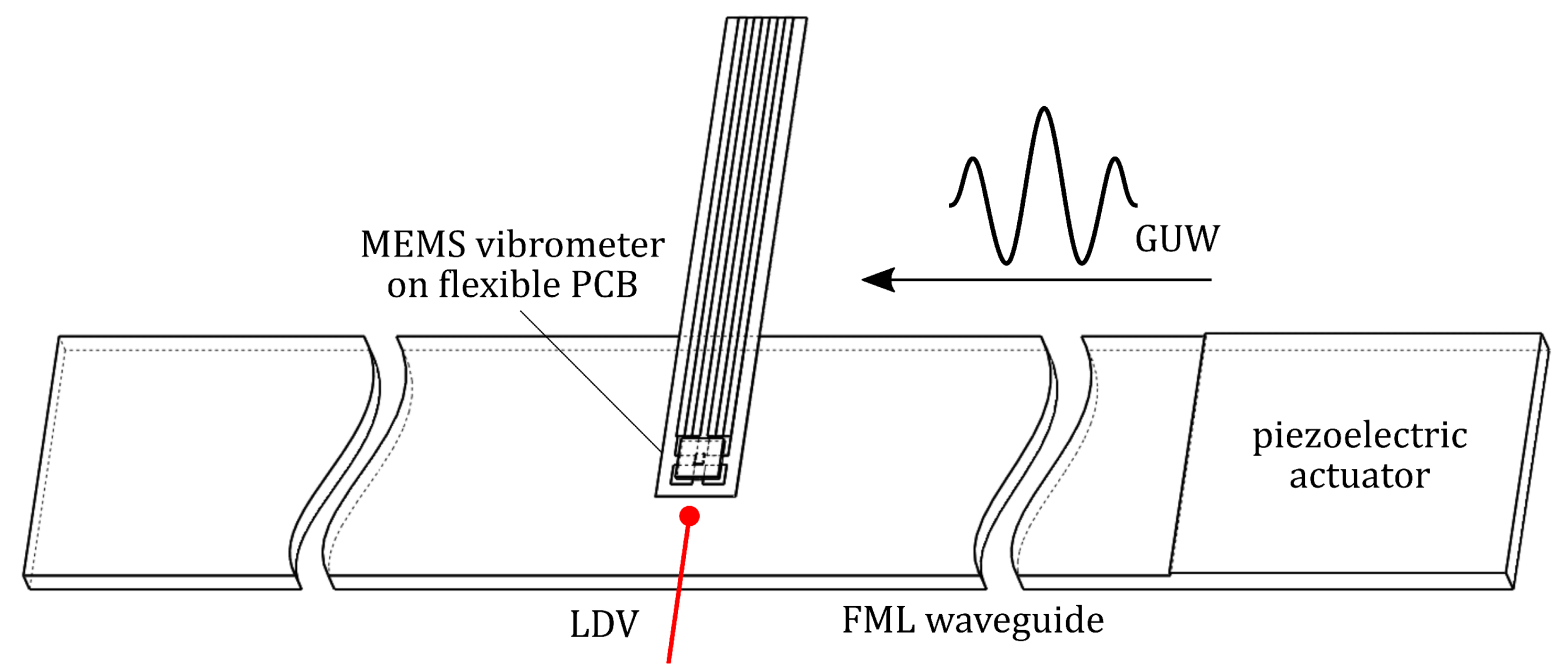
Test setup for GUW sensing experiments: FML waveguide with surface-bonded piezoceramic actuator, applied MEMS vibrometer, and reference LDV measurement at a distance of 0.2 m from excitation.

The MEMS vibrometer is mounted onto a 0.5 m long FML strip to demonstrate the functionality of sensing GUWs. The strip is from CFRP and steel with the stacking sequence ${\left[\mathrm{steel}/0_{4}/\mathrm{steel}/0_{2}\right]}_{S}$ (in the nomenclature common for layered composite materials) with a nominal thickness of 2.02 mm. Ultrasonic, hanning-windowed sinusoidal bursts are excited at one edge using a rectangular piezoceramic with a thickness of 0.2 mm, cf. Figure 7. The thereby-excited waves propagate through the wave guide. At 0.2 m of distance from the excited edge, the MEMS vibrometer is mounted. In addition to the electrical response, the out-of-plane velocity is measured by the LDV measuring directly next to the sensor.

## 4. Results

### 4.1. Modal Analysis of the Micro Cantilever

**Figure 8:**
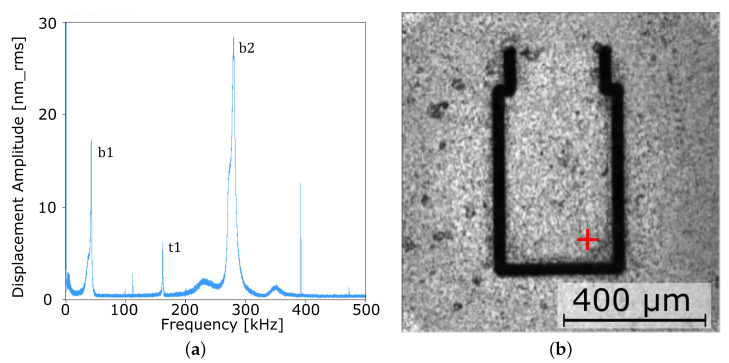
(**a**) Vibrational response of the micro cantilever measured at the location indicated by the red mark in the microscopy image (**b**). Peaks b1, b2, and t1 denote bending and torsional modes.

Figure 8a shows the amplitude response of the cantilever up to 500 kHz as obtained with the SmarAct PicoScale vibrometer at the indicated (red mark) location in Figure 8b. Three major resonances appear at 42 kHz, 162 kHz, and 281 kHz. Parasitic peaks at 99 kHz, 112 kHz, 393 kHz, and 473 kHz originate from the electronics of the PicoScale vibrometer.

**Figure 9:**
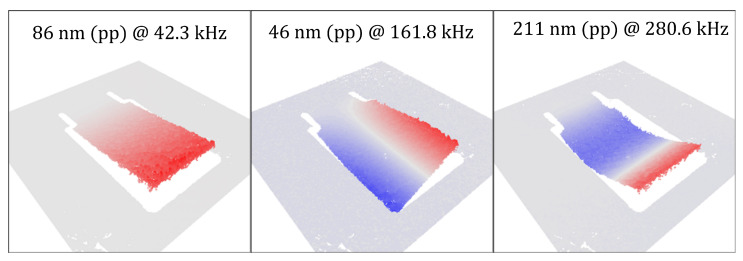
Representation of the bending (**left**, b1 and **right**, b2) and torsional (**center**, t1) modes recorded with the laser scanning vibrometer. Red and blue coloring represents the normalized deflection. The amplitudes shown correspond to the peak-to-peak values.

The images obtained with the PicoScale vibrometer (Figure 9) show the shapes of bending and torsion modes up to a frequency of 500 kHz. The obtained bending resonances are in sufficient agreement with the analytical predictions.

### 4.2. Dynamic MEMS Vibrometer Response

**Figure 10:**
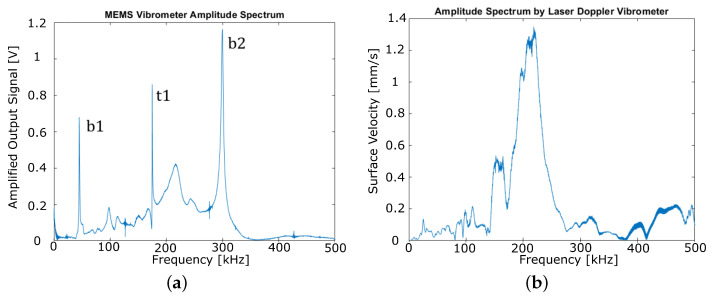
(**a**) Frequency response of the MEMS vibrometer chip as obtained by the setup shown in Figure 6. Bending (b1, b2) and torsional modes (t1) are indicated. (**b**) Velocity amplitude as obtained by the LDV measurement.

Figure 10a shows the electrical signal amplitude spectrum of the MEMS vibrometer between 0 and 500 kHz as obtained on the piezoceramic shaker stage (Figure 6). Narrow resonances can be recognized around 50 kHz, 180 kHz, and 300 kHz which fit quite well to both the previously calculated and measured mechanical resonances of the micro cantilever. In addition, some broader peaks are found in the spectrum that are influenced by GUW reflections and resonance phenomena in the measurement setup. To verify this, the complex velocity spectrum, as obtained by the LDV between 0 and 500 kHz, is divided by iω to obtain the displacement spectrum of the shaker stage the MEMS vibrometer is applied on. Figure 10b depicts the amplitude spectrum of the shaker stage’s displacement. Even though the LDV spot could not be exactly placed on the MEMS chip frame, this measurement can be regarded as approximately representing the frequency characteristics of the sensor’s mechanical excitation. As expected, the LDV spectrum reveals a dynamic behavior of the shaker stage that appears in the MEMS signal as well, e.g., the broad peak at approximately 220 kHz. However, the sharp peaks in the sensor signal do not appear in the shaker stage’s displacement spectrum such that they can be clearly identified as the sensor’s resonances.

### 4.3. Detection of GUW

To demonstrate the MEMS vibrometer’s ability to acquire GUW, ultrasonic sine bursts are excited into an FML wave guide. These are then recorded by the MEMS vibrometer and an LDV at the approximately same position but on the wave guide. Unlike the experimental setup in Figure 6, this configuration in Figure 7 allows the ultrasonic wave package to propagate through the FML wave guide before it is picked up by the LDV and the MEMS vibrometer.

**Figure 11:**
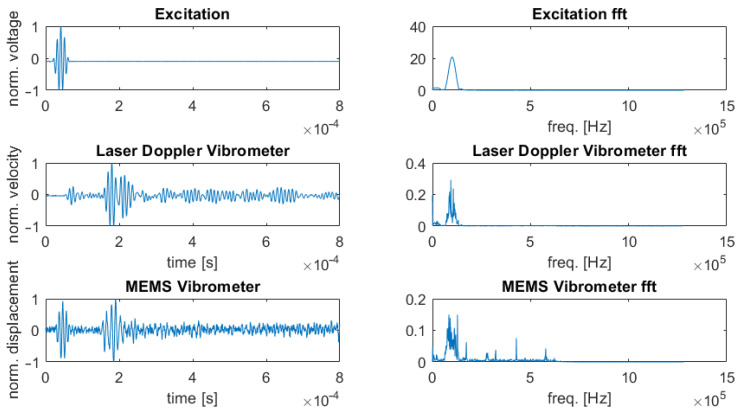
Comparison of signals of GUW in time and frequency domain. **Top**: Excitation signal (output of function generator). **Middle**: Signal acquired with the LDV with $S_{0}$ wave package at t≈70 μs and $A_{0}$ wave package at t≈180 μs. **Bottom**: Signal of the MEMS vibrometer with electrical cross-talk at t≈50 μs and $A_{0}$ wave package at t≈180 μs.

In Figure 11, the signals of excitation (top), LDV (middle), and MEMS vibrometer (bottom) are displayed. The LDV signal depicts a first wave package at t≈ 70 μs and a second one at t≈ 180 μs. Considering the excitation of the GUW at t≈ 50 μs and the distance traveled to be 0.2 m, this results in group velocities of approximately $c_{g}\left(S_{0}\right)\approx 6650\;{\mathrm{ms}}^{-1}$ and $c_{g}\left(A_{0}\right)\approx 1450\;{\mathrm{ms}}^{-1}$ for the first and second wave package. This is in sufficient agreement with the theoretically expected group velocities which result as $c_{g}\left(S_{0}\right)=7155\;{\mathrm{ms}}^{-1}$ and $c_{g}\left(A_{0}\right)=1367\;{\mathrm{ms}}^{-1}$ with the help of GUIGUW [20], a tool for the computation of acoustic wave dispersion features for the composite structure described in Section 3.4. Another indicator supporting this conclusion is the signal amplitude. The first measured wave package, corresponding to the $S_{0}$-mode, has a lower amplitude than the second one. The LDV measures the structural velocity in the laser direction which is nearly perpendicular to the specimen’s surface, resulting in a higher sensitivity for out-of-plane vibrations. In this frequency range, the $A_{0}$-mode has a higher out-of-plane proportion than the $S_{0}$-mode.

The MEMS vibrometer is equally capable of detecting two wave packages. However, the first one is due to an electrical cross-talk, meaning that the electrical excitation signal directly couples into the MEMS signal with no delay and covers a eventually occurring $S_{0}$ wave package. This should be avoidable with better electrical shielding. The second wave package is detected at t≈ 180 μs. This is again the $A_{0}$-mode with a higher out-of-plane component. This measurement is in good agreement with the working principle of the MEMS vibrometer as it is designed to be sensitive in the out-of-plane direction.

These measurements validate the concept of an MEMS vibrometer for detecting the out-of-plane component of GUW. At frequencies lower than the first resonance, a sensitivity to acceleration exists, but more importantly a sensitivity to displacements can be concluded between the first bending and the first torsional resonance and therefore between 40 kHz and 160 kHz.

## 5. Discussion

An MEMS vibrometer is presented that can be embedded in the layered structure of FML and can capture information from inside the structure without disturbing the propagating GUW. The MEMS vibrometer is based on a mechanical spring-loaded mass oscillator that can sense dynamic displacement fields of structure-borne ultrasound beyond its first eigenfrequency. The dynamic behavior of the spring mass system was investigated by means of micro-LSV, and eigenmodes could be identified. Between the first bending and the first torsional resonance, i.e., between 40 kHz and 160 kHz, a sensitivity to displacement was confirmed. The sensor was therefore ideally suited for receiving GUW bursts with a center frequency of 100 kHz.

We demonstrated this capability using a GUW setup with an FML strip as wave guide. The MEMS vibrometer could record GUW that had traveled over 0.2 m distance to their excitation. The signals were in good agreement with those obtained with an LDV positioned in the proximity of the MEMS vibrometer. Due to its non-isotropic sensitivity, out-of-plane displacements can be selectively recorded and GUW mode selectivity can be achieved even for a single point of interest.

The presented MEMS vibrometer is encapsulated in a glass chip package and can be embedded into a composite structure in a next step to capture ultrasonic displacement fields of the inner material layers. Preliminary embedding experiments could already demonstrate the successful integration of MEMS vibrometers into both glass laminate aluminum reinforced epoxy (GLARE) and CFRP–steel plates.

In future work, an improved resonator design with a higher sensitivity and a larger bandwidth will be presented. In contrast to an accelerometer, where $|{\underline{G}}_{accel}|\approx m\;\cdot \;k^{-1}$, the sensitivity of the vibrometer can only be increased by improving the piezoresistive transmission, e.g., by maximizing the strain response in the piezoresistive path and by choosing a piezoresistive material with a high k-factor. Possible manufacturing tolerances would lead to small variations of the eigenfrequencies. The overall performance, however, would not be affected as long as the signal is within the frequency band, with $|{\underline{G}}_{displ}|\approx 1$.

A characterization of the sensitivity and the signal-to-noise-ratio will be addressed, using a tailored characterization environment with longer propagation distance that was very recently established [21]. Further, sensor damping adjustment will be possible by entrapping gas of defined pressure inside the sensor cavity. GUW-based SHM will be performed using FML-embedded MEMS vibrometers with improved resonators. In future work, an integrable sensor data acquisition node [22], will be used to wirelessly power the vibrometer and read its GUW signals. 

## Data Availability

The raw data of the experiments can be requested from the authors.
